# Retrograde Versus Orthograde Obturation in Relation to Root Resection: Evaluation of Microhardness of Mineral Trioxide Aggregate In Vitro

**DOI:** 10.1155/ijod/6647418

**Published:** 2025-12-02

**Authors:** Lukas Stundžia, Rita Vėberienė, Indrė Graunaitė, Aurelijus Domeika, Neringa Skučaitė

**Affiliations:** ^1^Clinic of Dental and Oral Pathology, Medical Academy, Lithuanian University of Health Sciences, Kaunas, Lithuania; ^2^Institute of Mechatronics, Kaunas University of Technology, Kaunas, Lithuania

**Keywords:** microhardness, mineral trioxide aggregate, MTA, orthograde obturation, retrograde obturation

## Abstract

Limited visibility of the operating field can lead to difficulties in relation to adequate retrograde filling during periapical surgery in specific clinical situations. Solid and homogenic root filling material that does not need to be removed after apicoectomy could be used as an alternative to retrograde filling. This study aims to compare the micro-hardness of mineral trioxide aggregate (MTA) considering retrograde vs. orthograde obturation during root end resection in vitro. Methods: 20 roots of maxillary incisors were chemo-mechanically prepared and divided into 2 groups: group A (*n* = 10)—retrograde filling and group B (*n* = 10)—orthograde filling. The specimens of group A were filled with gutta-percha and resin-based sealer, 3 mm of the apical area were removed after the incubation period. Retrograde cavities of a depth of 3 mm were made and filled with MTA. In group B, apical 7 mm of the roots was filled with MTA in an orthograde manner. After the incubation, 3 mm of the root ends were removed. Vickers microhardness test was applied to the MTA No statistically significant difference in micro-hardness between groups was found (*p* > 0.05). The assessment of retrograde filling did not reveal significant differences of MTA micro-hardness in different parts of the material (*p* > 0.05). The assessment of orthograde filling revealed that MTA micro-hardness 3 mm from the apex was significantly higher when compared to the distance of 1 and 2 mm from the root apex (*p* > 0.05). The obturation method did not affect the hardness of MTA during root end resection in vitro.

## 1. Introduction

Since its introduction in the late 1990s, mineral trioxide aggregate (MTA) has become the preferred material in apical surgery as root-end filling cement, showing excellent long-term results [1]. Although new hydraulic calcium silicate cements have been introduced recently, MTA remains the material of choice for endodontic surgery. Numerous studies reported characteristics of this cement eligible for root-end filling: MTA is biocompatible, able to set in the presence of moisture, stimulates hard tissue formation, has an antibacterial effect due to high pH values, provides better sealing and marginal adaptation [2–7].

MTA is hydrophilic cement, nevertheless, has long initial setting time (2 h 45 min) [8]. In the surgical site with excess bleeding, there is always a risk that freshly mixed MTA will be washed out. An excess of moisture and bleeding can affect adhesion, hardness, and surface morphology of the material [9–12]. On the contrary, some studies showed that introduction of moisture had no significant effect on the cement after 3 min of its setting [13]. Microhardness testing could be suggested to evaluate the quality of the cement's setting. Another drawback of MTA is poor handling properties [8]. Surgical access can be compromised by anatomic structures, anatomy of teeth or insufficient mouth opening in clinical situations when root end resection is the best option of endodontic treatment. The retro preparation and retro filling could be complicated and time consuming in such cases due to limited visibility of the operating field.

MTA, as well as other calcium silicate cements may be used as a root canal obturation material in cases with wide apical foramen, either naturally in young patients or due to pathological root resorption [14]. Root canals filled with MTA before periapical surgery might be beneficial due to compromised surgical access in clinical situations when root end resection is planned and the possibility of endodontic treatment before surgical procedure exists. Moreover, orthograde obturation is preferred in cases when endodontic treatment is required for teeth that will be involved during a cystectomy procedure eliminating the need for retrograde treatment during surgery and preserving the length of involved roots. Furthermore, preparing the roots before surgery reduces the time and difficulty of the surgical procedure and as such should be considered in all cases if orthograde obturation is possible. Thus, it is important to know whether MTA used as an orthograde filling material will maintain its mechanical properties following the resection, and if the hardness of orthograde MTA will be similar to the retrograde filling of the apical part of the root.

The aim of this study was to identify and compare the microhardness of MTA with respect to the orthograde vs. retrograde method used to fill the apical part of the root in case of the root end resection in vitro.

## 2. Materials and Methods

Before beginning the experiment, approval was received from the Lithuanian University of Health Sciences Bioethics Community (Number 2024-BEC3-T-004).

### 2.1. Specimens' Preparation

Twenty freshly extracted human maxillary incisors were selected for the study. The teeth were evaluated under a dental microscope (Carl Zeiss, Germany) for any cracks, caries, or any other defects. The teeth were decoronated, leaving the roots 15 mm of length. The root canals were shaped using rotary instruments ProTaper Gold Universal (Dentsply Sirona) to the size F5 (50/05) at the apex. Irrigation during instrumentation was performed with 2.5% sodium hypochlorite and finalized with 5 mL of sterile water. All specimens divided into groups according to the method used to fill the apical part of the root with MTA (ProRoot MTA, Dentsply Sirona): group A (*n* = 10)—retrograde, group B (*n* = 10)—orthograde.

Group A: the root canals were filled with gutta-percha and resin-based sealer (AH Plus, Dentsply Sirona); orifices were isolated using temporary restorative material IRM (Dentsply Sirona). After incubation at 37 ± 1°C and 100% humidity for 72 h apical parts of obturated roots (3 mm) were cut off perpendicular (90°) to the long axis of the root using diamond bur. Retro cavities were prepared at 3 mm depth by ultrasonic tips KiS-2D (Kerr Dental) and filled with MTA. Filling material was mixed according to the manufacturer's instructions for 30 s to produce a homogenous mixture. Machtou hand pluggers (Dental VDW) were used to condense MTA into the retro cavities.

Group B: Apical parts of roots (7 mm) were filled with MTA in orthograde manner using Machtou hand pluggers for condensation of the material; a wet paper point was inserted intracanal over MTA, orifices of the roots were isolated with temporary restorative material IRM.

**Figure 1:**
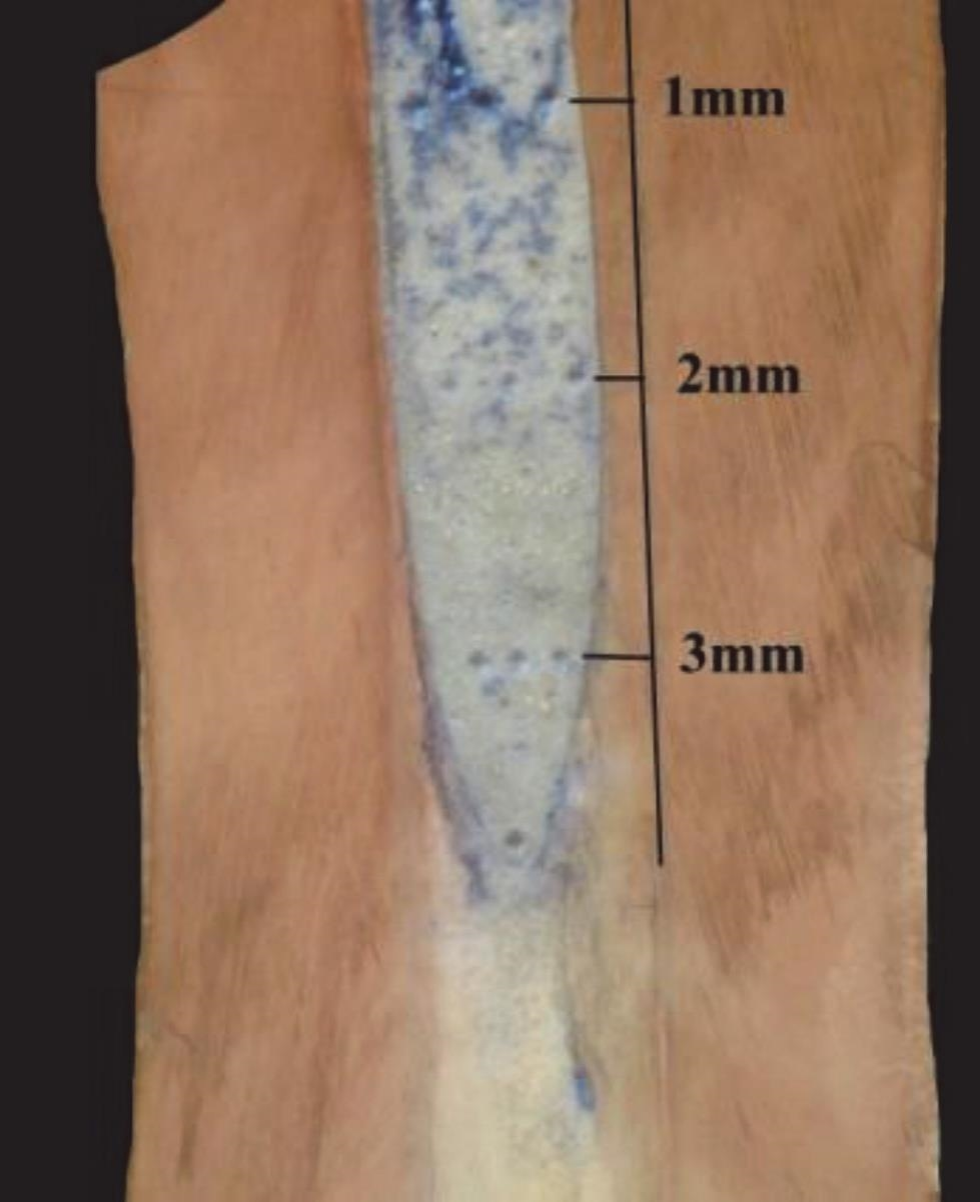
Division of the longitudinally sectioned resected root with retrograde MTA obturation (group A) prepared for the measurements of the material microhardness.

**Figure 2:**
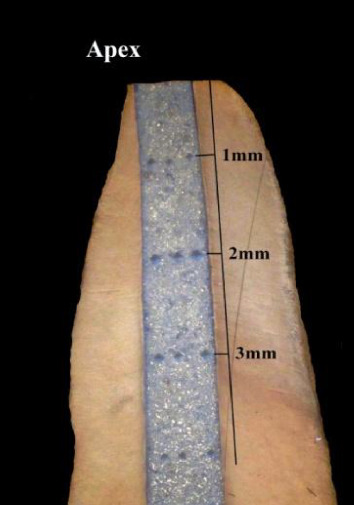
Division of the longitudinally sectioned resected root filled with MTA in orthograde manner (group B) prepared for the measurements of the material microhardness.

Specimens of both groups after obturation with MTA were embedded into a sponge soaked with saline to simulate physiologic conditions; stored at 37 ± 1°C, 100% humidity for 72 h to allow setting of MTA. Apices of roots (3 mm) in group B were cut off perpendicular (90°) to the long axis of the root after incubation; the remaining part of MTA in the canal was kept as retro-filling (Figures 1 and 2).

All procedures were performed by one experienced operator using a dental microscope (Carl Zeiss, Germany).

### 2.2. Microhardness Testing

All specimens were subjected to the Vickers microhardness test. The roots were embedded with acrylic resin using a prosthetic liner to ensure orientation in the long axis of the root, slightly wet-polished longitudinally uncovering the middle part of MTA with a polishing machine (LAM PLAN SMARTLAM 2.0); using various sizes of abrasive paper discs (Lam Plan). The specimens were then cleaned, gently washed, and dried by air spray. A clinical hand probing test was done on each specimen to confirm MTA setting. Then the Vickers microhardness test was performed at every millimeter (3 mm) of each specimen. The longitudinal sections of the roots were divided into millimeters under the microscope control (Carl Zeiss, Germany). Microhardness of the cement was measured by indenting the polished surface with 136° tip of the diamond pyramid. A full load of 200 g was applied to the surface of the cement for 30 s at room temperature. Three indentations were produced at every millimeter of the surface of MTA (Figures 1 and 2). Thus, microhardness of MTA was measured at every millimeter of the obturation length of the specimens in both test groups (ortho and retro obturation), and the mean values of microhardness measured on three indentations at every surface millimeter were calculated.

The Vickers microhardness was calculated according to the formula:

  $$\begin{array}{c} \text{HV}=\frac{2F\;\sin\left(\frac{136{}^{\circ}}{2}\right)}{d^{2}},\text{ HV}=1.854\;\frac{F}{d^{2}}, \end{array}$$

*F* = load/kg; *d* is the mean of the two diagonals of the impression made by the intender in millimeters.

### 2.3. Statistical Analysis

Statistical analysis included the paired Student's *t*-test and Wilcoxon test to compare the mean microhardness values at every millimeter within the test group. A dispersion analysis ANOVA and Kruskal–Wallis tests were used to compare the MTA microhardness between the groups. The power of the study was selected to be *β* = 0.8, and the confidence level—*α* = 0.05.

## 3. Results

**Figure 3:**
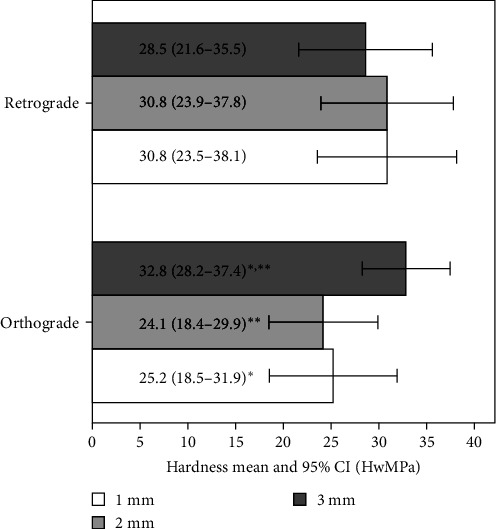
Mean surface microhardness values of MTA in the experimental groups (distance of 1, 2, and 3 mm from the resected root apex). *⁣*^*∗*^, *⁣*^*∗∗*^ significant differences *p*  < 0.05.

The mean surface microhardness values of MTA retrograde filling (group A) and orthograde filling (group B) at the distance of 1, 2, and 3 mm from the resected root apex, respectively, are presented in Figure 3. No significant differences were observed between the mean values of MTA surface microhardness when comparing every millimeter of the material length in group A (retrograde obturation) (*p* > 0.05). When the MTA microhardness was compared within the group B (orthograde obturation), the mean value estimated at 3 mm from the root apex after resection was significantly higher than those at 1 and 2 mm from the apex after resection (*p*  < 0.05). No statistically significant differences comparing mean surface microhardness values of MTA at every millimeter between the groups were found (*p* > 0.05).

## 4. Discussion

Obturating the entire root canal with MTA was suggested to simplify the apicoectomy procedure in cases when the possibility of endodontic treatment before root resection exists [14, 15]. Even though retrograde root canal obturation during periapical surgery and orthograde obturation only yield similar results concerning the properties of the apical plug of MTA [7, 10, 13, 16–18], it is important to ensure that the material will set evenly through the entire orthograde obturation length and the properties of MTA will not be altered following root-end resection.

Surface hardness is a measure of the relative set of the material. It is used for evaluating the quality and progression of the hydration process and serves as an indicator for the stability of the crystal structure of MTA and overall strength of the cement [19, 20]. The influence of the thickness of MTA on the quality of the apical seal has also been reported in the literature [21, 22]. A 7 mm thick MTA sample was used in this study, in regard to Harinkhere et al. [23], who stated that minimum 7–10 mm of an orthograde MTA filling is recommended to maintain appropriate apical seal after root resection. Furthermore, it is evident that minimum 4 mm of the obturation material should remain in the canal after resection of the apical part. In order to simulate clinical conditions, the samples were embedded in a saline soaked sponge to create moisture found in the periodontium. According to Shokouhinejad et al. [22], 6 mm thick MTA samples require two side moisture for a better hydration process, and there is no difference in using water or phosphate-buffered saline-moistened pellets. As such, a water-moistened paper point was placed intracanal over MTA in the orthograde obturation group to ensure the setting of material by creating two side moisture in this study. The samples were stored in 100% humidity for 72 h due to previous recommendations to allow MTA to set for at least 72 h before the Vickers test [19].

According to the results of this study, the hardness values of both orthograde and retrograde MTA groups were lower, when compared to the results reported by other authors, where they varied from 37.54 [24] to 99.82 MPa [25]. Although the data concerning the surface hardness of resected MTA is limited, the discrepancies of the results could be explained in different methodologies, amounts, and thicknesses of MTA used in these studies. Despite this, the clinical probing test, which was performed on every specimen before the Vickers test, showed completely hardened material. The pressure force generated by hand probing is not described in the literature, as this is the only method for evaluating the hardness of the cement in clinical practice.

The tendency to harden better near the source of moisture was also observed in this study. Though there was no significant difference, the apical 1st, 2nd mm of retrograde MTA samples that were covered with a sponge soaked with saline, was slightly harder than the 3rd mm from the apical part. In contrast, in the orthograde MTA group, the 3rd mm that was in direct contact with wet paper point was significantly harder compared to the 1st, 2nd mm from the root end resection side. It could be speculated that under clinical conditions, the orthograde MTA obturation uncovered during root end resection could be less hard in the apical part compared to retrograde filling due to less moisture reaching the middle part of the orthograde MTA during material setting. Nevertheless, no significant differences comparing surface microhardness of MTA at every millimeter between retrograde and orthograde MTA groups following root end resection were estimated in this study. This indicates that surface hardness of MTA could be similar regardless the ortho vs. retro root-end obturation method applied during apicoectomy procedure.

According to the study of Angerame et al. [26] following root-end resection the apical 3 mm of MTA used as obturation material showed no significant differences in the number of voids formed regardless of whether retrograde or orthograde obturation methods were used. Considering these findings, research concerning MTA microhardness [24, 25] and results of this in vitro study, it could be suggested that MTA can be used as a root canal filling material before apical surgery. Sato et al. [27] stated that even bone graft used during surgery before setting can influence properties of MTA. It could be assumed that the excess of moisture or bleeding that could adversely affect physiochemical properties of unhardened MTA would be avoided by resecting the roots obturated with MTA before surgical procedures in cases when the option of endodontic treatment exists [10, 11].

This study, as well as other in vitro studies concerning MTA, suffer from the fact that the final quality of MTA's physical properties relating to the material's mixing and condensation during obturation is highly dependent on the skill of the operator. Furthermore, the question of whether hardness testing can reflect optimal physiochemical properties of the material and prevention of microleakage remains unanswered.

Based on the results of this study and published results previously [16, 26, 28, 29] there is no difference in microhardness, sealing ability, or volume of voids between retrograde and orthograde root canal obturation following root end resection when MTA is used as filling material. In view of these findings, further clinical research concerning the outcomes of periapical surgery is needed to confirm the hypothesis and establish an alternative protocol of apicoectomy and root end filling procedure.

## 5. Conclusion

Within the limitation of this study, the microhardness of MTA was not affected by the different filling methods (orthograde vs. retrograde) used for obturation when root resection was performed. These findings are in line with available literature and confirm the validity of MTA use in endodontic surgical procedures. Furthermore, the findings indicate that in a clinical setting, obturating root canals with MTA root end resection procedures can be performed without adverse effects on the material regardless of the chosen obturation method.

## Data Availability

The data that support the findings of this study are available from the corresponding author upon reasonable request.
